# Prooxidative Activity of Celastrol Induces Apoptosis, DNA Damage, and Cell Cycle Arrest in Drug-Resistant Human Colon Cancer Cells

**DOI:** 10.1155/2019/6793957

**Published:** 2019-08-14

**Authors:** Helena Moreira, Anna Szyjka, Kamila Paliszkiewicz, Ewa Barg

**Affiliations:** Department of Basic Medical Sciences, Wroclaw Medical University, Borowska 211 street, 50-556 Wroclaw, Poland

## Abstract

Cancer resistance to chemotherapy is closely related to tumor heterogeneity, i.e., the existence of distinct subpopulations of cancer cells in a tumor mass. An important role is assigned to cancer stem cells (CSCs), a small subset of cancer cells with high tumorigenic potential and capacity of self-renewal and differentiation. These properties of CSCs are sustained by the ability of those cells to maintain a low intracellular reactive oxygen species (ROS) levels, via upregulation of ROS scavenging systems. However, the accumulation of ROS over a critical threshold disturbs CSCs—redox homeostasis causing severe cytotoxic consequences. In the present study, we investigated the capacity of celastrol, a natural pentacyclic triterpenoid, to induce the formation of ROS and, consequently, cell death of the colon cancer cells with acquired resistant to cytotoxic drugs (LOVO/DX cell line). LOVO/DX cells express several important stem-like cell features, including a higher frequency of side population (SP) cells, higher expression of multidrug resistant proteins, overexpression of CSC-specific cell surface marker (CD44), increased expression of DNA repair gene (PARP1), and low intracellular ROS level. We found that celastrol, at higher concentrations (above 1 *μ*M), significantly increased ROS amount in LOVO/DX cells at both cytoplasmic and mitochondrial levels. This prooxidant activity was associated with the induction of DNA double-strand breaks (DSBs) and apoptotic/necrotic cell death, as well as with inhibition of cell proliferation by S phase cell cycle arrest. Coincubation with NAC, a ROS scavenger, completely reversed the above effects. In summary, our results provide evidence that celastrol exhibits effective cytotoxic effects via ROS-dependent mechanisms on drug-resistant colon cancer cells. These findings strongly suggest the potential of celastrol to effectively kill cancer stem-like cells, and thus, it is a promising agent to treat severe, resistant to conventional therapy, colon cancers.

## 1. Introduction

Reactive oxygen species (ROS) are highly reactive, oxygen-containing, chemical molecules produced intracellularly through multiple mechanisms. The major endogenous sources of ROS are NADPH oxidase (NOX) complex in the cell membrane, mitochondria, peroxisomes, and endoplasmic reticulum. At low and moderate levels, cellular and mitochondrial ROS are implicated in various important cellular processes such as proliferation, differentiation, and survival. Excessive ROS levels interfere with redox homeostasis leading to a significant modification of the structure and function of cellular macromolecules that determine the fate of the cell. Importantly, chronically increased endogenous ROS is linked to the adaptive changes in cells that lead to cellular transformation and tumorigenesis [1–5]. Compared to normal cells, cancer cells display higher levels of ROS as a result of higher energy metabolism rate, oncogene activation, and loss of tumor suppressors [2, 6]. For instance, Haklar et al. have reported significantly increased levels of all ROS, especially hypochlorite, NO, and peroxynitrite in cancerous colon tissues [7]. Despite elevated intracellular ROS levels, cancer cells are sensitive to oxidative stress and ROS amplification over a critical threshold selectively kill tumor cells [8]. Most cytotoxic effects of chemotherapy are associated with the induction of cellular ROS generation. However, chronical exposure of cancer cells to ROS induced by chemotherapeutics, such as doxorubicin, daunorubicin, epirubicin, or camptothecin, leads to the development of drug-resistant phenotype which is associated with the overexpression of ATP-dependent transmembrane efflux pumps and reduced ROS level [9, 10].

Resistance to chemotherapy is closely related to tumor heterogeneity, i.e., the existence of distinct subpopulations of cancer cells in a tumor mass. An important role is assigned to cancer stem cells (CSCs), a small subset of cancer cells with high tumorigenic potential and capacity of self-renewal and differentiation. Compared to differentiated cancer cells, CSCs are quiescent and present lower energy metabolism rate that consequently results in a significantly lower level of basal ROS [4]. This is also achieved by upregulation of ROS scavenging systems such as glutathione (GSH) [11]. In gastrointestinal CSCs, increased intracellular GHS synthesis is maintained by CD44v, a cellular adhesion molecule. CD44v is overexpressed in CSC and is a critical regulator of cancer stemness, including self-renewal, tumor initiation, and metastasis [12, 13]. A growing amount of evidence indicates that CSCs and non-CSCs can be bidirectionally converged, i.e., non-CSCs can be reprogrammed into CSCs and conversely CSCs into non-CSCs phenotypes [8, 12]. In view of these abilities of cancer cells, anticancer therapy strategies should target both bulk differentiated cells and CSCs. The treatment that impairs ROS defense and/or induces ROS generation provides a potential approach for killing CSCs.

Celastrol (tripterine) is a natural polyphenolic compound; one of the most biologically active product isolated from the Celastraceae family plants. Celastrol has been shown to exhibit important antioxidant and anti-inflammatory activities. It also inhibits the secretion of proinflammatory cytokines. In the past decade, it has also been found to inhibit tumor proliferation and growth in various cancer models as well as tumor capacity to metastasis. Anticancer properties of celastrol arise from its pleiotropic activities on multiple cellular signal pathways, including multidrug resistance mechanisms [14–18]. It has been reported that celastrol synergistically enhances the cytotoxicity of radiotherapy and chemotherapeutic agents [19]. We have also demonstrated that celastrol was able to enhance the sensitivity of the doxorubicin-resistant colon cancer cells via direct binding to P-glycoprotein1 (P-gp), a multidrug resistance protein belonging to ATP-dependent transporters. Interestingly, a few recent reports have indicated that celastrol has the potential to induce intracellular ROS generation in lung, osteosarcoma, melanoma, and ovarian cancer cells [20–23]. This prooxidative activity of celastrol was associated with the induction of cytotoxic effects in these tumors. In addition, Seo et al. showed that celastrol augmented ROS production induced by ionizing radiation in lung cancer as a result of celastrol-induced thiol reactivity of antioxidant enzymes [24].

In this work, for the first time, the effect of celastrol on ROS amounts in colon cancer cells displaying a high level of resistance to cytotoxic drugs (LOVO/DX cell line) was investigated.

These cells express several important features of CSCs, such as increased frequency of side population (SP) cells, high efflux capacity through ATP-dependent transporters (mainly P-gp glycoprotein), overexpression of CD44 and PARP1, a DNA repair gene, and significantly lower intracellular ROS level. We demonstrated that celastrol was able to generate oxidative stress in LOVO/DX cells at both cytoplasmic and mitochondrial levels. This prooxidant activity was associated with the induction of ROS-dependent DNA double-strand breaks (DSBs), the strongly deleterious and harmful DNA damages. The high level of phosphorylated H2A.X histone (*γ*-H2A.X), a biomarker of DSBs, was related to inhibition of cell proliferation by S phase cell cycle arrest and induction of apoptotic cell death in these cancer cells. Coincubation with N-Acetyl-L-cysteine (NAC), a ROS scavenger, completely reversed above effects. These results provide evidence that celastrol exhibits effective cytotoxic effects via ROS-dependent mechanisms on drug-resistant colon cancer cells.

## 2. Materials and Methods

### 2.1. Materials

DMEM F12 (Dulbecco's Modified Eagle's Medium: Nutrient Mixture F-12), HBSS (Hank's Balanced Salt Solution), FBS (fetal bovine serum), ultraglutamine 1, and gentamicin sulfate were purchased from Lonza (Basel, Switzerland). Accutase™ Cell Detachment and FITC Annexin V Apoptosis Detection Kit were obtained from BD Biosciences (Franklin Lakes, New Jersey, USA). TrypLE™ Express was from Gibco (Waltham, MA, USA). MitoPy1 [4-[4-[3-Oxo-6′-(4,4,5,5-tetramethyl-1,3,2-dioxaborolan-2-yl)spiro [isobenzofuran-1(3H), 9′-[9H]xanthen]-3′-yl]-1-piperazinyl]butyl]triphenyl-phosphonium iodide) was from Tocris Bioscience (Bristol, United Kingdom). Bovine serum albumin (BSA), DCF-DA (2,7-dichlorofluorescin diacetate), NAC (N-Acetyl-L-cysteine), DMSO (Dimethyl Sulfoxide), paraformaldehyde (PFA), propidium iodide, and DAPI (4′,6-Diamidino-2-phenylindole dihydrochloride) were purchased from Sigma-Aldrich (St. Louis, MO, USA). Ethanol 96% was from Chempur. Celastrol, with purity more than 98%, was purchased from Cayman Chemical Company (Ann Arbor, MI, USA). Phospho-histone H2A.X (Ser139) monoclonal antibody (CR55T33) Alexa Fluor 488 eBioscience™ was obtained from Invitrogen (Carlsbad, CA, USA).

### 2.2. Methods

#### 2.2.1. Cell Line and Culture Conditions

The doxorubicin-resistant colon cell line (LOVO/DX) was derived from LOVO cell line (ATCC collection) by 3-month cultivation in the presence of a low concentration of doxorubicin. LOVO cell line originates from the metastatic site of colon adenocarcinoma. The LOVO/DX cells were cultured in DMEM F12 medium supplemented with 10% FBS, 2 mM L-glutamine, and 25 *μ*g/ml of gentamicin at 37°C in a humidified atmosphere with 5% CO_2_. The cells were subcultured twice a week using TrypLE™ Express.

### 2.3. Drug Solution

Celastrol was dissolved in DMSO as 10 mM stock solution and stored at -20°C. The working solution was freshly prepared before each experiment by 10x dilution of stock solution in a culture medium. The final DMSO concentration in the cell culture did not exceed 0.02%.

### 2.4. Detection of Intracellular ROS

The DCF-DA assay was carried out with flow cytometry according to the protocol previously described [25, 26]. The working DCF-DA solution was freshly prepared before each experiment by dissolving in 100% ethanol and further 10x dilution in HBSS. The final DCF-DA concentration in cell culture was 20 *μ*M.

The LOVO/DX cells were removed from the culture flask using TrypLE™ Express solution, spun down, and pelleted. Cells (1 × 10^6^/ml) were then resuspended in 1 ml of freshly prepared DCF-DA/HBSS solution in plastic Falcon tubes, and celastrol was immediately added to the samples to the final concentrations of 0.1-20 *μ*M. The samples were incubated for 1 h at 37°C in a CO_2_ incubator. Following incubation time, the cells were washed in HBSS and stained with propidium iodide (PI) for dead cell exclusion from the analysis. Afterwards, all samples were placed on ice and immediately analyzed for intracellular content of DCF using flow cytometer.

### 2.5. Detection of Mitochondrial H_2_O_2_

The mitochondria peroxy yellow 1 (MitoPY1), a fluorescent probe that selectively tracks to the mitochondria, was used to measure mitochondrial hydrogen peroxide (H_2_O_2_) [27]. MitoPY1 was dissolved in DMSO as 5 mM stock solution and aliquoted into PCR tubes. The aliquots (20 *μ*l) were placed in a desiccator under weak vacuum until the solvent was removed and then stored at -20°C. Working solution (10 *μ*M) was freshly prepared before each experiment by dissolving the aliquot in 20 *μ*l of DMSO and then by adding 10 ml of HBSS.

The LOVO/DX cells were removed from the culture flask using TrypLE™ Express solution, spun down, and pelleted. Then, the cell pellets (1 × 10^6^ cells/sample) were resuspended in 1 ml of freshly prepared MitoPY1 solution in plastic Falcon tubes. Afterwards, celastrol solution was immediately added to the cells to the final concentrations of 0.1-20 *μ*M. The samples were incubated for 1 h at 37°C in a CO_2_ incubator and then washed in HBSS. For dead cell exclusion from analysis, the cells were costained with propidium iodide (PI). The cell-associated fluorescence was measured by flow cytometry.

### 2.6. Apoptosis and Necrosis Assay

Apoptosis and necrosis were detected with flow cytometry after staining of the cells with fluorochrome mixture: Annexin V-FITC and PI, using the FITC Annexin V Apoptosis Detection Kit. The staining allows to discriminate between early and late apoptotic cells and necrotic cells. LOVO/DX cells (7.5 × 10^5^/ml) were seeded in a 12-well plate and incubated with various celastrol concentrations in the absence or presence of 5 mM NAC (N-Acetyl-L-cysteine) (37°C, 5% CO_2_). Following 4 hours incubation, the cells were detached with Accutase™ Cell Detachment and washed with HBSS. The cells were resuspended in 100 *μ*l of ice-cold 1x binding buffer and stained with 5 *μ*l of Annexin V-FITC and 5 *μ*l of PI for 15 minutes in the dark at room temperature. Samples were immediately analyzed with the flow cytometer.

### 2.7. Cell Cycle Analysis

The flow cytometric analysis of cell cycle was done by the means of propidium iodide (PI) staining according to the protocol described in the literature [28]. The PI staining solution was freshly prepared before each experiment and contained PI 0.1% (*v*/*v*) Triton X-100, 50 *μ*g/ml PI (Molecular Probes Inc.), and 50 *μ*g/ml DNase-free RNase A in PBS.

LOVO/DX cells (1 × 10^6^/ml) were seeded in a 6-well plate and incubated with various celastrol concentrations (37°C, 5% CO_2_). Following 18 hours of incubation, the cells were removed from wells using TrypLE™ Express solution and washed with cold HBSS. The cells were then fixed with ice-cold 70% ethanol and kept on ice for 1 hour. After two washing steps with cold HBSS, the cells were resuspended in 0.5 ml of PI staining solution and incubated for 30 minutes in the dark. Samples were then analyzed with the flow cytometer.

### 2.8. Detection of *γ*-H2A.X

Detection and quantification of *γ*-H2A.X^+^ positive cells were done using phospho-histone H2A.X (Ser139) monoclonal antibody (CR55T33) Alexa Fluor 488, based on the protocol previously described by Kataoka et al. [29].

LOVO/DX cells (1 × 10^6^/ml) were seeded in a 6-well plate and incubated for 4 hours with various celastrol concentrations (37°C, 5% CO_2_). Then, the cells were removed with TrypLE™ Express solution, placed into plastic Falcon tubes, and washed twice with cold HBSS. The cells were fixed using 2% paraformaldehyde (PFA) for 10 minutes on ice. After two washing steps with cold HBSS containing 1% bovine-serum albumin (1% BSA-HBSS), the cells were permeabilized with ice-cold 70% ethanol (prepared in 1% BSA-HBSS). The samples were kept in this solution for 5-7 days at 4°C. Before staining with antibody, the cells were washed twice using 1% BSA-HBSS. The cell pellets were resuspended in 100 *μ*l of 1% BSA-HBSS containing 2 *μ*l of phospho-histone H2A.X (Ser139) monoclonal antibody (CR55T33) Alexa Fluor 488 and incubated for 30 minutes in the dark. Then, the cells were washed with 1% BSA-HBSS and counterstained with DAPI (1 *μ*g/ml) for cell cycle analysis. Stained cells were analyzed by flow cytometry.

### 2.9. Flow Cytometric Analysis

In all assays, the cells were acquired on CyFlow® SPACE flow cytometer (Sysmex, Kobe, Prefektura Hyōgo, Japan). The laser excitation 488 nm (50 mW) and the filter 536/40 (BP) were used for fluorescence measurement of DCF, MitoPY1, FITC, and Alexa Fluor 488. Propidium iodide fluorescence was measured using laser excitation 488 nm (50 mW) and 675/20 (BP) filter and DAPI fluorescence with 375 nm (16 mW) laser excitation and 455/50 (BP) filter. The results were analyzed using FlowMax (Sysmex, Kobe, Prefektura Hyōgo, Japan) or FCS express 4 flow software (De Novo Software, Glendale, CA, USA). The MultiCycle™ DNA analysis model was used for cell cycle analysis.

### 2.10. Statistical Analysis

Statistical significance of the results was calculated using GraphPad Prism Version 6.05 (GraphPad Software, La Jolla, CA, USA).

## 3. Results

### 3.1. Intracellular ROS Level in LOVO/DX Cells

**Figure 1:**
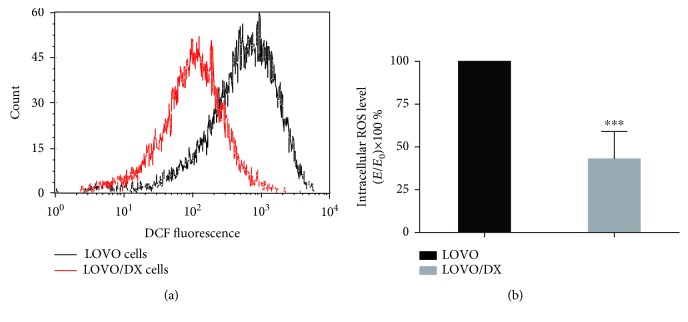
Intracellular ROS level (DCF-DA assay) in LOVO/DX cells compared to LOVO cells. (a) Representative histograms of flow cytometric evaluation of the cell-associated DCF fluorescence. (b) The basal level of intracellular ROS in LOVO/DX cells compared to LOVO cells. Results are expressed as *E*/*E*_0_ × 100% (mean ± SD, *n* = 5, ^∗∗∗^ *p* ≤ 0.0001), where the MFI (mean fluorescent intensity) of DCF in LOVO/DX cells (*E*) was compared to the MFI in LOVO cells (*E*_0_).

Several reports indicate that drug-resistant cancer cells and CSCs present a reduced amount of ROS than drug-sensitive cancer cells. Therefore, we first evaluated the basal ROS level in LOVO/DX cells exhibiting high resistance to doxorubicin compared to sensitive cells (LOVO cells). Intracellular ROS amount was assessed by flow cytometry using DCF-DA assay. As shown in Figure 1, the endogenous ROS level in LOVO/DX cells is significantly lower (>50%) than in LOVO cells.

### 3.2. Effect of Celastrol on ROS Level in LOVO/DX Cells

**Figure 2:**
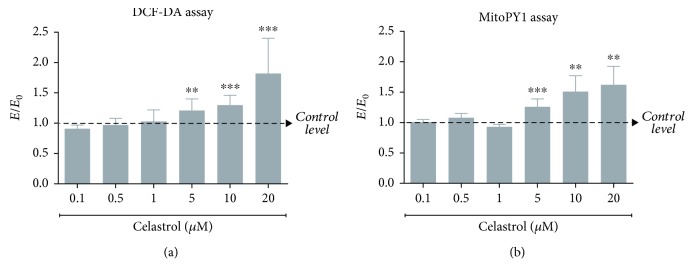
Impact of celastrol on the intracellular ROS level (DCF-DA assay) (a) and the mitochondrial H_2_O_2_ level (MitoPY assay) (b) in LOVO/DX cell cultures. The results obtained in the presence of celastrol (*E*) were compared to the relevant control (*E*_0_), i.e., cells incubated in the presence of the solvent (DMSO). The values are expressed as the mean ± SD, *n* = 6, ^∗∗^ *p* ≤ 0.01, ^∗∗∗^ *p* ≤ 0.001.

To investigate whether celastrol is able to induce an increase in the amount of endogenous ROS in drug-resistant cells, the LOVO/DX cells were treated with various concentrations of celastrol. Since mitochondria are the major intracellular source of ROS, mitochondrial H_2_O_2_ content (MitoPY1 assay) was evaluated in addition to cytosol ROS amount (DCF-DA test). The influence of celastrol on ROS and H_2_O_2_ generation is shown in Figures 2(a) and 2(b), respectively. The results indicate that ROS levels did not change or slightly decreased following cell incubation with lower celastrol concentrations (0.1-1 *μ*M). However, at higher concentrations (5-20 *μ*M), it induces significant ROS accumulation at both cytosol and mitochondrial levels. This increase is dose-dependent and reaches up to 80% (DCF-DA) and 60% (MitoPY1) above the control level, at the highest concentration of 20 *μ*M.

### 3.3. Cytotoxic Effect of Celastrol on LOVO/DX Cells

**Figure 3:**
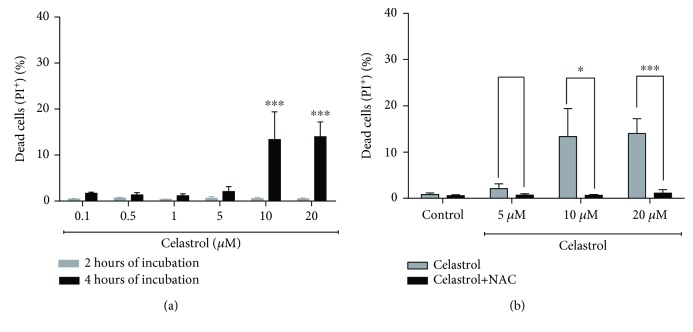
The frequency of necrotic cells in LOVO/DX cell cultures incubated with celastrol (a) and coincubated with celastrol and NAC (b). The cells were double stained with Annexin V-FITC and propidium iodide (PI) fluorescent dyes (FITC Annexin V Apoptosis Detection Kit) and analyzed by flow cytometry. Control: LOVO/DX cells incubated without celastrol (gray), LOVO/DX cells incubated without celastrol and NAC (black). The results are presented as a percentage of PI^+^ cells (Annexin V-FITC^−^ and PI^+^ dead cells); mean ± SD, *n* = 4, ^∗^ *p* < 0.05, ^∗∗∗^ *p* ≤ 0.001.

It is well known that excessive ROS production can affect the viability of cancer cells. Therefore, we investigated whether celastrol prooxidative activity could lead to cytotoxic effects in LOVO/DX cells. The celastrol-induced cytotoxicity was determined after 2 and 4 hours of the treatment by means of PI staining. In shorter incubation time, celastrol had no impact on LOVO/DX cell viability at the tested concentration range. Extending the incubation time to 4 hours led to the appearance of necrotic cells in a very small percentage (1.2–2%) at lower concentrations. At the higher concentrations, 10 and 20 *μ*M, the percentage of necrotic cells increases up to 14% (Figure 3(a)). This effect is completely abolished in cell culture incubated with celastrol in the presence of ROS scavenger: *N*-acetylcysteine (NAC, 5 mM) (Figure 3(b)).

### 3.4. Effects of Celastrol on Apoptotic Cell Death of LOVO/DX Cells

**Figure 4:**
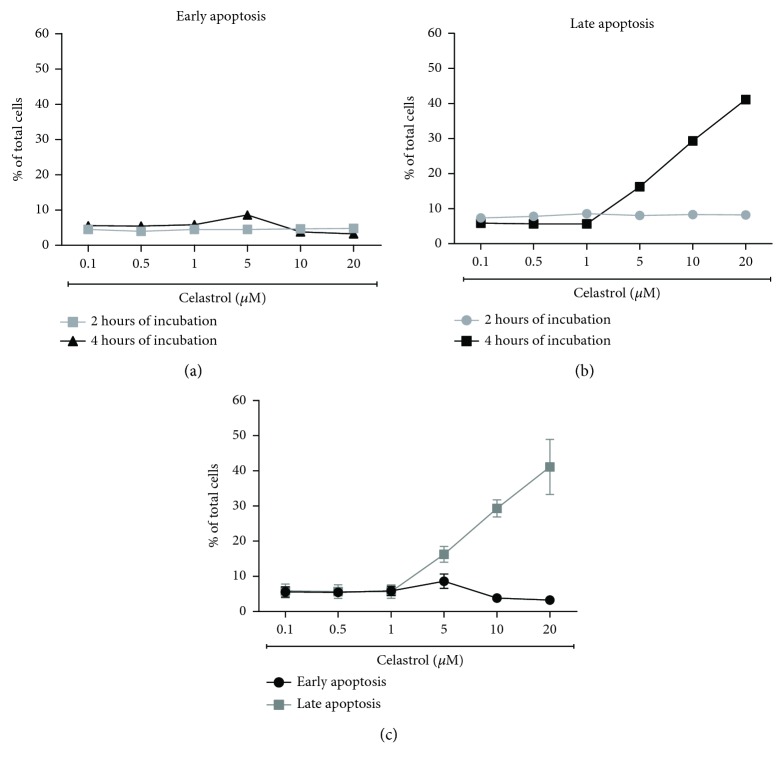
The effects of 2 and 4 hours of incubation of celastrol with LOVO/DX cells on the frequency of early and late apoptosis. The cells were double stained with Annexin V-FITC and PI fluorescent dyes (FITC Annexin V Apoptosis Detection Kit) and analyzed by flow cytometry. The results are presented as a percentage of early apoptotic cells (Annexin V-FITC^+^ and PI^−^) and late apoptotic cells (Annexin V-FITC^+^ and PI^+^); mean ± SD, *n* = 4.

**Figure 5:**
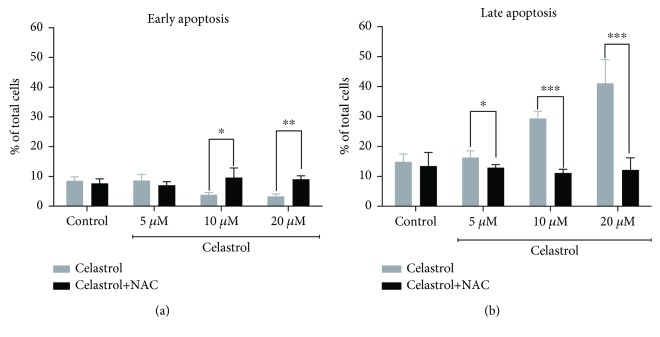
The frequency of early and late apoptotic cells in LOVO/DX cell cultures incubated with celastrol (a) and coincubated with celastrol and NAC (b). The cells were double stained with Annexin V-FITC and PI fluorescent dyes (FITC Annexin V Apoptosis Detection Kit) and analyzed by flow cytometry. Control: LOVO/DX cells incubated without celastrol (gray), LOVO/DX cells incubated without celastrol and NAC (black). The results are presented as a percentage of early apoptotic cells (Annexin V-FITC^+^ and PI^−^) and late apoptotic cells (Annexin V-FITC^+^ and PI^+^); mean ± SD, *n* = 4, ^∗^ *p* < 0.05, ^∗∗^ *p* ≤ 0.01, and ^∗∗∗^ *p* ≤ 0.001.

**Figure 6:**
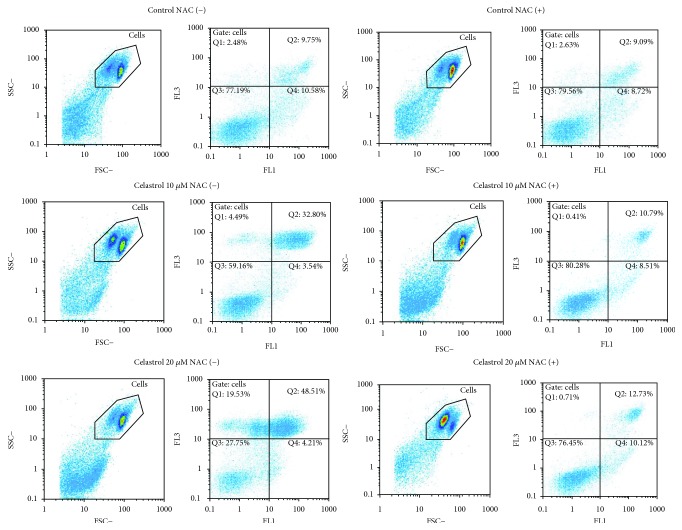
Flow cytometric analysis of early and late apoptosis in LOVO/DX cell cultures incubated with celastrol or coincubated with celastrol and NAC. Representative cytograms are shown. FSC = forward light scatter, SSC = side light scatter, FL1 = Annexin V-FITC, FL3 = PI, Q1 = necrotic cells (Annexin V-FITC^−^ and PI^+^), Q2 = late apoptotic cells (Annexin V-FITC^+^ and PI^+^), Q3 = live cells (Annexin V-FITC^−^ and PI^−^), Q4 = early apoptotic cells (Annexin V-FITC^+^ and PI^−^).

ROS and mitochondria play an important role in apoptosis induction. We evaluated whether the prooxidant activity of celastrol is associated with apoptosis induction in LOVO/DX cells. Proapoptotic effects of celastrol were studied after 2 and 4 hours of incubation with LOVO/DX cells using double staining with Annexin V-FITC and PI dye. The results are presented as a percentage of early apoptotic cells (Annexin V-FITC^+^ and PI^−^) and late apoptotic cells (Annexin V-FITC^+^ and PI^+^). As shown in Figures 4(a) and 4(b), the frequency of cells with early and late apoptotic features does not change after 2 hours of incubation regardless of the celastrol concentration. However, longer incubation time leads to a significant increase in the percentage of late apoptotic cells up to 41% at 5–20 *μ*M concentrations of celastrol (Figure 4(b)). Moreover, there is a clear disproportion between the number of cells in early and late stages of apoptosis, i.e., the decrease of early apoptotic cells along with the increase of late apoptotic cells is observed (Figure 4(c)). The addition of NAC (5 mM) to the cell cultures entirely abolishes these effects of celastrol (Figures 5 and 6).

### 3.5. Impact of Celastrol on Cell Cycle and DNA Damage

**Figure 7:**
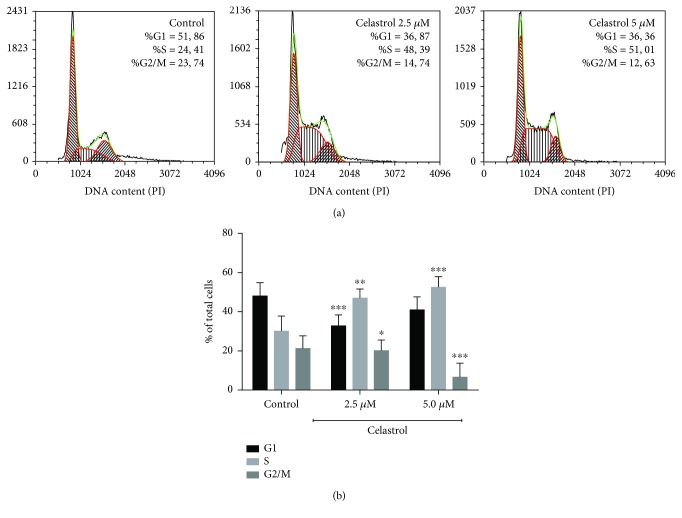
Impact of celastrol on cell cycle distribution in LOVO/DX cells. (a) Representative histograms of flow cytometric analysis of cell cycle. (b) Bar graph showing the percentage of LOVO/DX cells in each cell cycle phase after incubation with solvent (DMSO) or celastrol. The results are presented as mean ± SD, *n* = 4, ^∗^ *p* < 0.05, ^∗∗^ *p* ≤ 0.01, and ^∗∗∗^ *p* ≤ 0.001.

**Figure 8:**
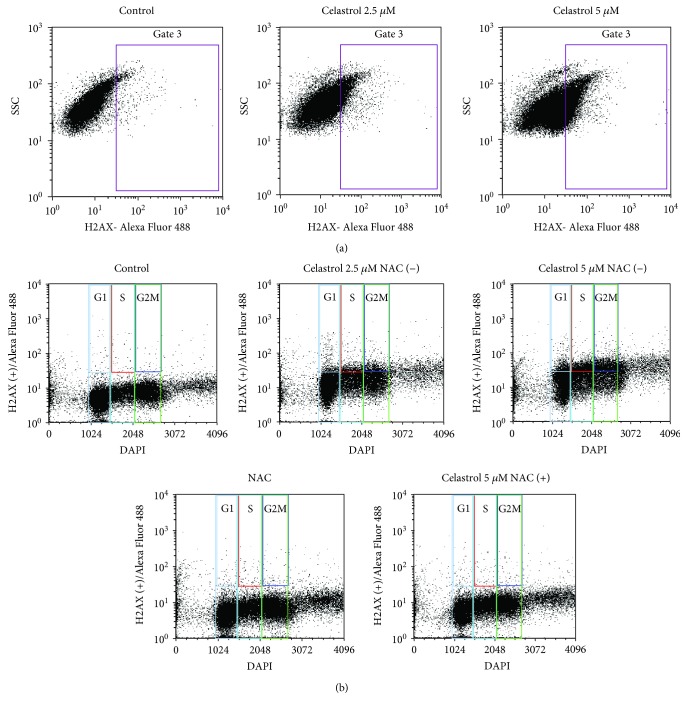
Representative histograms of flow cytometric analysis of DNA damages (*γ*-H2AX^+^ cells) and cell cycle phases in LOVO/DX cells after treatment with celastrol. (a) Detection of *γ*-H2AX^+^ cells using phospho-histone H2A.X (Ser139) monoclonal antibody (CR55T33) Alexa Fluor 488. SSC = side light scatter. (b) Bivariant cell cycle/DAPI-combined *γ*-H2AX analysis.

In addition to apoptosis induction, ROS, at higher levels, cause oxidative DNA damage and subsequent cell cycle arrest. We thus investigated whether celastrol-induced apoptosis was associated with inhibition of cell cycle and induction of double-strand breaks in LOVO/DX cells. The cell cycle was evaluated by PI staining.  Figure 7(a) depicts the distribution of LOVO/DX cells through the cell cycle after treatment with celastrol for 18 hours. The results reveal that celastrol causes cell cycle arrest by the accumulation of cells in the S phase together with a marked reduction in the number of cells in the G2/M phase (Figure 7(b)). Moreover, at 2.5 and 5 *μ*M of celastrol, a marked increase in the generation of double-strand breaks was observed, as assessed by *γ*-H2AX (Table 1, Figure 8(a)). *γ*-H2A.X^+^ positive cells were observed in all phases of the cell cycle; however, S phase cells revealed a lower frequency of *γ*-H2A.X^+^ cells compared to G2/M phase cells (Table 2, Figure 8(b)). The addition of NAC (5 mM) to the cell culture incubated with celastrol results in the complete abolishment of *γ*-H2AX formation (Tables 1 and 2).

## 4. Discussion

Reactive oxygen species (ROS) play an important role in the anticancer activity of several agents used for the treatment of colon and other cancers. Amplification of the intracellular ROS level in tumor cells induces apoptosis and, in some cases, other types of cell death, i.e., autophagy or necrosis by damaging cellular components such as DNA and protein and lipid membranes. Recently, some naturally occurring polyphenols have been reported to act as selective cytotoxic agents against cancer cells by generation of toxic levels of ROS [30]. Celastrol is a plant triterpenoid that strongly inhibits the growth and development of cancer in various cancer cell models. Some molecular mechanisms responsible for its anticancer activity have been proposed. The celastrol structure holds a highly redox-active para-quinone methide moiety that can induce oxidative stress by forming ROS, such as superoxide or hydrogen peroxide [31, 32]. Some recent reports have indicated that celastrol induces antitumor effects by increasing the intracellular accumulation of ROS in lung, osteosarcoma, melanoma, and ovarian cancer cells [20–24]. However, the effect of celastrol on drug-resistant, stem-like colon cancer cells is still unexplored.

In the present study, we aimed to determine whether celastrol is able to induce the formation of ROS and consequently cell death in colon cancer cells with acquired resistant to cytotoxic drugs. For this purpose, we used human colon adenocarcinoma cell line (LOVO/DX) which shows cross-resistance to doxorubicin and other anthracyclines such as vinca alkaloids, epipodophyllotoxin derivatives, 4′-(9-acridinylamino-methanesulfon-*m*-aniside), and actinomycin D [33]. We compared the endogenous redox status of those cells with their sensitive counterpart (LOVO) and we found that LOVO/DX cells have a significantly lower level of cytoplasmic ROS. Maiti has reported that ovarian cancer cells resistant to chlorambucil (A2780/100) present reduced amount of ROS compared to sensitive cells and that decreased ROS level is one of the main reasons for developing and maintaining the resistance of those cells. In addition, the elevation of the cellular ROS by exogenous ROS generation increases the A2780/100 sensitivity [10]. In our previous paper, we have shown that celastrol exhibits significant chemopreventive and chemosensitizing activities on LOVO/DX cells, in part by inhibition of P-gp, a multidrug-resistant protein [34]. Although we demonstrated that celastrol has the ability to bind directly to P-gp, we hypothesized that ROS generation might be an additional mechanism by which celastrol exerts its anticancer effects in those cells. Indeed, celastrol was able to generate a significant amount of intracellular ROS at both cytoplasmic and mitochondrial level. The prooxidant activity of celastrol was limited to higher concentrations—above 1 *μ*M. This is in agreement with literature data indicating that phenolic compounds can act as a prooxidant only under certain conditions, i.e., at elevated doses. In addition, celastrol-induced generation of ROS was significantly decreased by ROS scavenger, *N*-acetylcysteine (NAC) (Figure S1, supplementary data).

Cellular ROS, and particularly mitochondrial H_2_O_2_, have been identified as critical intermediates in the activation of the apoptotic process via the mitochondria-dependent and mitochondria-independent pathways [35]. Chen et al. demonstrated that in lung cancer cells (H1299 cell line) celastrol induced ROS generation by inhibiting the activity of complex I MRC and not by inhibiting the expression of antioxidant proteins. Thus, ROS accumulation in H1299 cells was associated with apoptotic and necrotic cell death by downstream activation of JNK and downregulating client proteins HSP90 [20]. In our study, celastrol was also able to induce apoptotic and, in a smaller extent, necrotic cell death in doxorubicin-resistant colon cancer cells. Those effects were only observed at prooxidative concentrations and appeared after 4 hours of incubation. In addition, hydrogen peroxide (H_2_O_2_), one of the most important ROS, induces apoptosis of LOVO/DX cells, in a dose-dependent manner (Figure S2, supplementary data). Moreover, the coincubation with NAC significantly decreased celastrol- and hydrogen peroxide-induced cell death. These observations point that the generation of ROS plays an important role in the apoptotic/necrotic activity of this phenolic compound.

ROS, at higher levels, are known to induce oxidative DNA damage and subsequent cell cycle arrest [1, 36]. Previously, it has been shown that celastrol was able to induce cell cycle arrest at the G_2_/M phase in ovarian cancer cells and at the G_0_/G_1_ phase in monocytic leukemia cells [23, 37]. In our study, we demonstrated that celastrol exerts growth-inhibitory effects via arresting the cell cycle at the S phase in doxorubicin-resistant cancer cells. The S phase is a crucial stage in the cell cycle progression as it allows for proper replication of DNA. Some anticancer drugs inhibit cell proliferation by inducing DNA double-strand breaks that result in cell cycle arrest at the S phase [36]. Increased level of intracellular free radicals, reacting with DNA and thereby modifying its structure and function, is one of the main causes of DNA damage [38]. Here, we showed that celastrol (at 2.5 and 5 *μ*M) significantly increases the quantity of *γ*-H2AX, a well-known marker of DNA damage, in LOVO/DX cells. The *γ*-H2AX formation showed no cell cycle-phase specificity and was completely abolished in the presence of NAC. Moreover, coincubation of celastrol (5 *μ*M) with NAC restored normal diploid distribution of LOVO/DX cells in the relevant cell cycle phases. These observations confirm the role of celastrol-induced ROS in the S phase cell cycle arrest and DNA damage. Interestingly, the increase in the *γ*-H2AX level was not observed at 20 *μ*M concentration of celastrol. Huang et al. have demonstrated that the drug-induced *γ*-H2AX appears very early during treatment, prior to caspase 3 activation, and decreases importantly at late stages of apoptosis which is characterized by increased levels of chromatin condensation [39]. In our study, celastrol at higher concentrations (10 and 20 *μ*M) caused a significant increase in the number of cells with late apoptotic features together with a decrease in the cell number bearing early apoptotic characteristics. Thus, *γ*-H2AX could not be detected in LOVO/DX cells after cell treatment with these doses.

Our data show that celastrol possesses a significant anticancer potential against drug-resistant colon cancer cells. This activity is related to the induction of DNA double-strand breaks, S phase cell cycle arrest, and triggering apoptosis. The increase in intracellular production of ROS induced by celastrol appears to be an important mechanism of its cytotoxic activity. ROS are highly reactive and react with DNA and proteins inducing cancer cell death. It should be emphasized that celastrol was able to cause anticancer effects only at higher concentrations in which it acts as a prooxidant. In addition, these doses do not change the viability of normal human cells, suggesting its specificity for tumor cells (Figure S3, supplementary data). It seems also that the mechanisms of celastrol cytotoxicity might differ in drug-sensitive and drug-resistant cancer since we found that in LOVO cells, celastrol mainly induces necrosis and has only a small proapoptotic effect (Figure S4, supplemented data). However, both this issue and the impact of celastrol on normal epithelial cells of the colon mucosa require further investigation.

In summary, we demonstrated that ROS play an important role in the cytotoxic activity of celastrol in resistant colon cancer cells. The resistance of colon cancer to chemotherapy is linked to the content of the cancer stem cells (CSCs) [34, 40]. Our previous study indicated that LOVO/DX cells contain an almost 7-fold greater number of CSC (measured by the size of the SP cell subpopulation) than the LOVO cells, ordinarily sensitive to cytostatics [34]. Moreover, celastrol-induced inhibition of P-gp function significantly lowered the SP fraction. The high efflux capacity through ATP-depending transporters is one of the most important CSC features that prevents the accumulation of cytostatic drug within the cells [11]. Interestingly, the LOVO/DX cells carry other stem-cell characteristics such as higher expression of CD44, a CSC-specific cell surface marker, and PARP1, a DNA repair gene (data not shown). Also, significantly lower ROS content was found in LOVO/DX cells compared to sensitive cells, as was mentioned above. In CSCs, the low amount of ROS sustains their self-renew potential and improves the abilities of invasion and resistance against therapy. CSCs maintain lower intracellular ROS level in part as a consequence of the modulation of the redox systems. For instance, in gastrointestinal CSCs, increased CD44v variant isoform expression contributes to ROS defense through GSH-dependent antioxidant mechanism [41]. Interestingly, Peng et al. have shown that celastrol has the ability to directly react with a thiol such as NAC and GSH (when coincubated) which results in reversing G0/G1 cell cycle arrest in U937 cells [37]. This finding strongly suggests that celastrol might induce cytotoxic effects not only by the direct increase of the ROS level but also via GSH depletion. Evidence to date has shown that despite the low ROS level and elevated antioxidant defense mechanisms in CSCs, accumulating ROS over a critical threshold that alters redox-homeostasis selectively kills these cells [8]. Taken together, it may be assumed that celastrol plays an important role in CSC clearance in drug-resistant colon cancer cells via a ROS-dependent mechanism.

## 5. Conclusions

Drug-resistant colon cancer cells possess a large number of CSC-specific features. We found that celastrol demonstrates prooxidative activity on those cells and causes ROS-dependent DNA DSBs which results in the expression of *γ*-H2A.X^+^. The ROS-induced DNA damage leads to inhibition of cell proliferation by S phase cell cycle arrest and induction of apoptotic cell death. These findings strongly suggest the potential of celastrol to effectively kill cancer stem-like cells, and thus, it is a promising agent to treat severe, resistant to conventional therapy, colon cancers. Further studies should be performed to confirm these results in *in vivo* models of colon cancer.

## Figures and Tables

**Table 1:** Effect of celastrol on the percentage of *γ*-H2AX^+^ cells in LOVO/DX cell culture.

	*γ*-H2AX^+^ cells (%)
Control	1.18 ± 0.39
CEL 1 *μ*M	3.37 ± 2.14
CEL 2.5 *μ*M	18.78 ± 6.65
CEL 5 *μ*M	26.93 ± 1.99
CEL 20 *μ*M	2.17 ± 1.39
NAC	1.54 ± 0.11
CEL 5 *μ*M+NAC	1.70 ± 0.62

**Table 2:** Effect of celastrol on the number of *γ*-H2AX^+^ cells in different phases of cell cycle in LOVO/DX cell culture.

	*γ*-H2AX^+^ cells in cell cycle phases (%)		
	G1	S	G2/M
CEL 1 *μ*M	0.29	0.18	0.32
CEL 2.5 *μ*M	***3.85***	***4.95***	***7.48***
CEL 5 *μ*M	***8.69***	***6.97***	***9.73***
CEL 20 *μ*M	0.11	0.12	0.13
NAC	0.37	0.36	0.38
CEL 5 *μ*M+NAC	0.58	0.53	0.57

## Data Availability

All data used to support the findings of this study are included within the article. The additional data, demonstrating the CD44 and PARP1 expression in LOVO/DX cells, are available from the corresponding author upon request.

## Supplementary Materials

Supplementary Materials Figure S1: impact of celastrol and celastrol coincubation with NAC on the intracellular ROS level (DCF-DA assay) in LOVO/DX cells. Representative histograms of flow cytometric evaluation of the cell-associated DCF fluorescence. Figure S2: flow cytometric analysis of early and late apoptosis in LOVO/DX cell cultures incubated with H_2_O_2_ and coincubated with H_2_O_2_ and NAC. The cells were incubated with H_2_O_2_ for 4 hours (37°C, 5% CO_2_) and then double stained with Annexin V-FITC and PI fluorescent dyes (FITC Annexin V Apoptosis Detection Kit). Representative cytograms are shown. FSC = forward light scatter, SSC = side light scatter, FL1 = Annexin V-FITC, FL3 = PI, Q1 = necrotic cells (Annexin V-FITC and PI^+^), Q2 = late apoptotic cells (Annexin V-FITC^+^ and PI+), Q3 = live cells (Annexin V-FITC and PI^−^), Q4 = early apoptotic cells (Annexin V-FITC^+^ and PI^−^). Figure S3: effect of celastrol on the viability of NHDF cells (normal human dermal fibroblast) (A) and PBMC cells (B). (A) The cells were incubated with celastrol for 4 hours (37°C, 5% CO_2_). The cell viability was measured by the means of XTT proliferation assay. The mean optical density (OD, absorbance (*A*)) was used to calculate the percentage of viable cell viability as follows: percentage of viable cells = (*A*_Celastrol_)/(*A*_control_) × 100%; mean ± SD, *n* = 5 (B). PBMC was activated with LPS and incubated with celastrol for 24 hours (37°C, 5% CO_2_). The viability of cells was assessed with the Guava PCA-96 Nexin Kit by flow cytometry; mean ± SD, *n* = 3 (data from PhD thesis: “Study of the Molecular Mechanisms TNF-*α* Secretion, a Key Cytokine in Chronic Inflammation” by Helena Tabaka-Moreira, Université de Strasbourg, France). Figure S4: the frequency of apoptotic and necrotic cells in LOVO cell cultures incubated with celastrol. The cells were incubated with celastrol for 4 hours (37°C, 5% CO_2_) and then double stained with Annexin V-FITC and PI fluorescent dyes (FITC Annexin V Apoptosis Detection Kit) and analyzed by flow cytometry. The results are presented as a percentage of early apoptotic cells (Annexin V-FITC^+^ and PI^−^), late apoptotic cells (Annexin V-FITC^+^ and PI^+^), and necrotic cells (Annexin V-FITC and PI^+^); mean ± SD, *n* = 5.
