# Supplementary material for: Prooxidative Activity of Celastrol Induces Apoptosis, DNA Damage, and Cell Cycle Arrest in Drug-Resistant Human Colon Cancer Cells
Source: Oxid Med Cell Longev. 2019 Aug 14;2019:6793957. doi: 10.1155/2019/6793957 (PMC6710751; doi:10.1155/2019/6793957)
Supplement: Supplementary Materials — Figure S1: impact of celastrol and celastrol coincubation with NAC on the intracellular ROS level (DCF-DA assay) in LOVO/DX cells. Representative histograms of flow cytometric evaluation of the cell-associated DCF fluorescence. Figure S2: flow cytometric analysis of early and late apoptosis in LOVO/DX cell cultures incubated with H2O2 and coincubated with H2O2 and NAC. The cells were incubated with H2O2 for 4 hours (37°C, 5% CO2) and then double stained with Annexin V-FITC and PI fluorescent dyes (FITC Annexin V Apoptosis Detection Kit). Representative cytograms are shown. FSC = forward light scatter, SSC = side light scatter, FL1 = Annexin V-FITC, FL3 = PI, Q1 = necrotic cells (Annexin V-FITC and PI+), Q2 = late apoptotic cells (Annexin V-FITC+ and PI+), Q3 = live cells (Annexin V-FITC and PI−), Q4 = early apoptotic cells (Annexin V-FITC+ and PI−). Figure S3: effect of celastrol on the viability of NHDF cells (normal human dermal fibroblast) (A) and PBMC cells (B). (A) The cells were incubated with celastrol for 4 hours (37°C, 5% CO2). The cell viability was measured by the means of XTT proliferation assay. The mean optical density (OD, absorbance (A)) was used to calculate the percentage of viable cell viability as follows: percentage of viable cells = (A Celastrol)/(A control) × 100%; mean ± SD, n = 5 (B). PBMC was activated with LPS and incubated with celastrol for 24 hours (37°C, 5% CO2). The viability of cells was assessed with the Guava PCA-96 Nexin Kit by flow cytometry; mean ± SD, n = 3 (data from PhD thesis: “Study of the Molecular Mechanisms TNF-α Secretion, a Key Cytokine in Chronic Inflammation” by Helena Tabaka-Moreira, Université de Strasbourg, France). Figure S4: the frequency of apoptotic and necrotic cells in LOVO cell cultures incubated with celastrol. The cells were incubated with celastrol for 4 hours (37°C, 5% CO2) and then double stained with Annexin V-FITC and PI fluorescent dyes (FITC Annexin V Apoptosis Detection Kit) and analyzed by flow c [file 6793957.f1.pdf]

Supplementary data

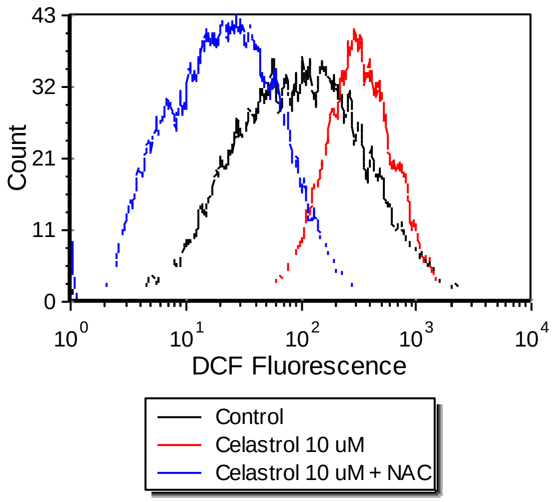

Figure 1S. Impact of Celastrol and Celastrol co-incubation with NAC on intracellular ROS level (DCF-DA assay) in LOVO/DX cells. Representative histograms of flow cytometric evaluation of the cell-associated DCF fluorescence.

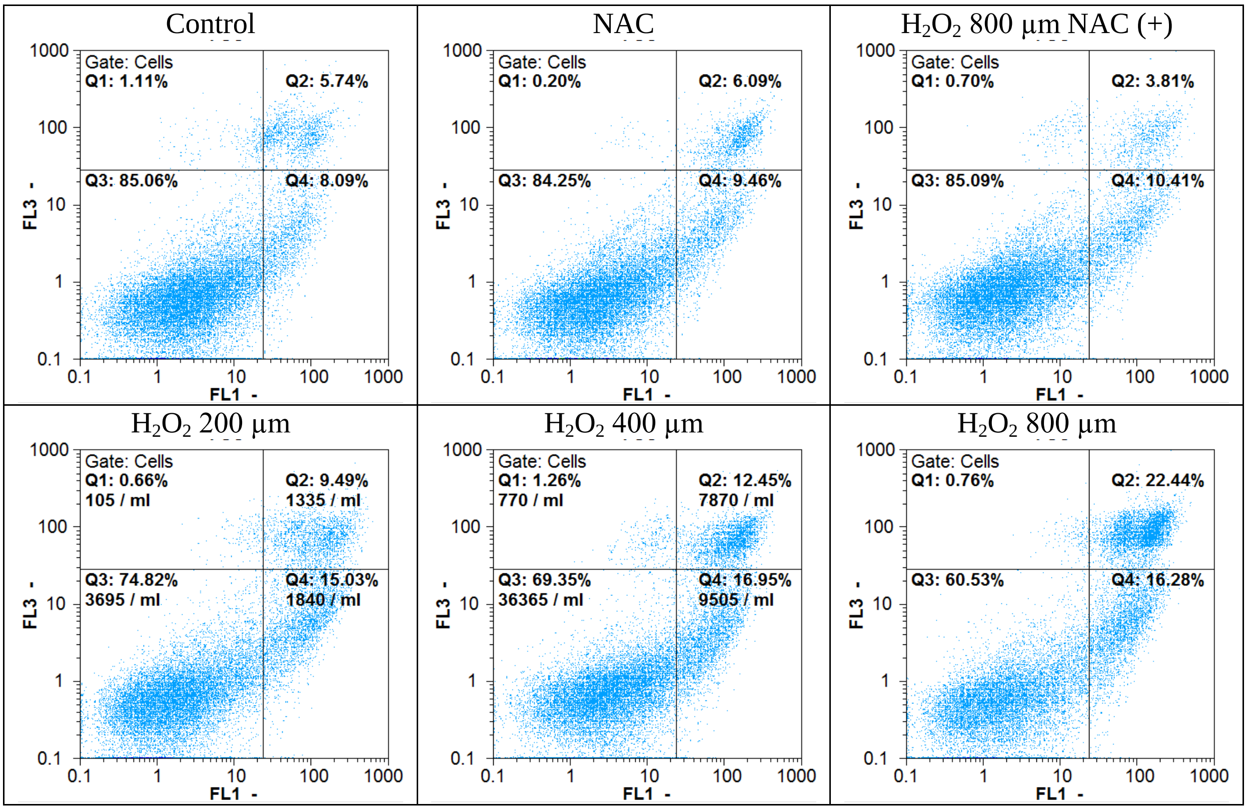

Figure 2S. Flow cytometric analysis of early and late apoptosis in LOVO/DX cell cultures incubated with H_2O_2 and co-incubated with H_2O_2 and NAC. The cells were incubated with $H_2O_2$for 4 hours (37°C, 5 % CO_2) and then double stained with Annexin V-FITC and PI fluorescent dyes (FITC Annexin V Apoptosis Detection Kit). Representative cytograms are shown. FSC=forward light scatter, SSC= side light scatter, FL1=Annexin V-FITC, FL3=PI, Q1= Necrotic cells (Annexin V-FITC$^-$ and PI$^+$), Q2= Late apoptotic cells (Annexin V-FITC$^+$ and PI$^+$), Q3=Live cells (Annexin V-FITC$^-$ and PI$^-$), Q4=early apoptotic cells (Annexin V-FITC$^+$ and PI$^-$)

[A]

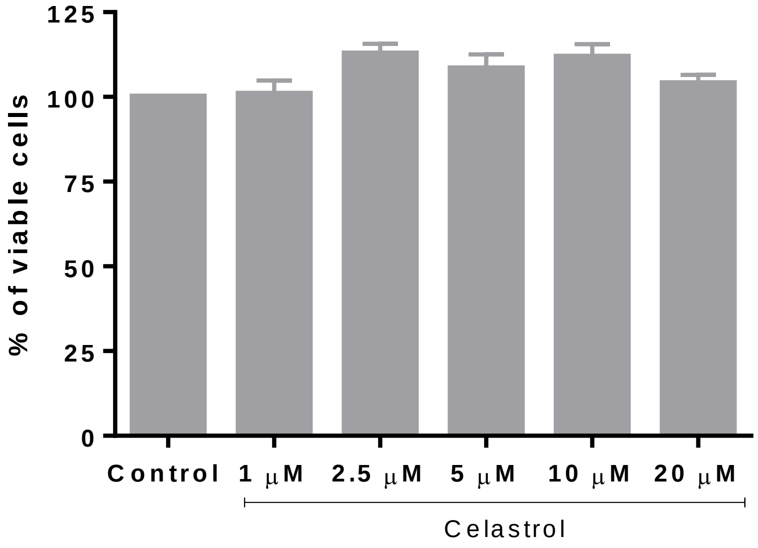

[B]

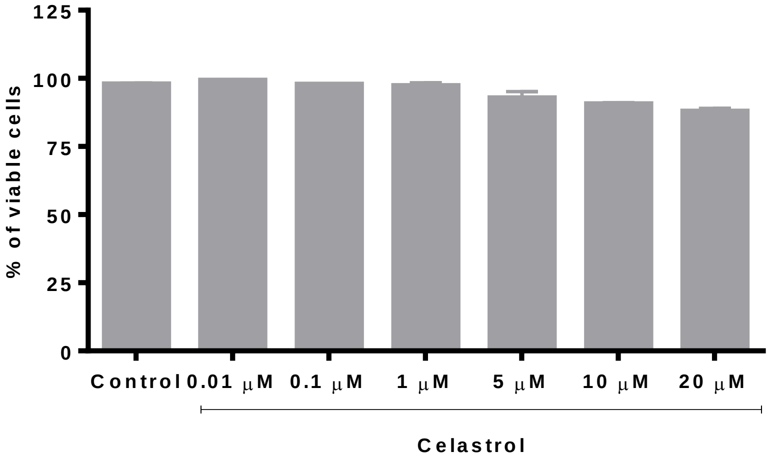

Figure 3S. Effect of celastrol on viability of NHDF cells (normal human dermal fibroblast) [A] and PBMC cells [B]. [A] The cells were incubated with celastrol for 4 hours (37°C, 5 % CO_2). The cell viability was measured by the means of XTT proliferation assay. The mean optical density (OD, absorbance (A)) was used to calculate the percentage of viable cell viability as follows (percentage of viable cells = $(A_{Celastrol})/(A_{control}) \times 100\%$. mean ± SD, n = 5

[B] PBMC were activated with LPS and incubated with Celastrol for 24 hours (37°C, 5 % CO_2). The viability of cells was assessed with Guava PCA-96 Nexin Kit by flow cytometry. mean ± SD, n = 3 (data from PhD thesis: "Study of the molecular mechanisms TNF-α secretion, a key cytokine in chronic inflammation" by Helena Tabaka-Moreira, Université de Strasbourg, France)

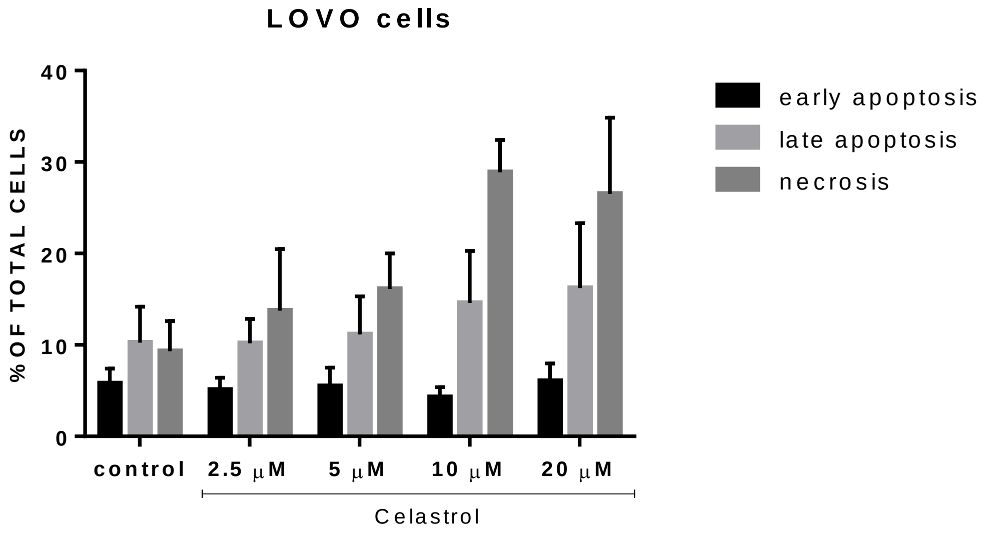

Figure 4S. The frequency of apoptotic and necrotic cells in LOVO cell cultures incubated with Celastrol. The cells were incubated with Celastrol for 4 hours (37°C, 5 % CO_2) and then double stained with Annexin V-FITC and PI fluorescent dyes (FITC Annexin V Apoptosis Detection Kit) and analyzed by flow cytometry. The results are presented as a percentage of early apoptotic cells (Annexin V-FITC$^+$ and PI$^-$), late apoptotic cells (Annexin V-FITC$^+$ and PI$^+$) and necrotic cells (Annexin V-FITC$^-$ and PI$^+$). mean ± SD, n = 5
